# Iatrogenic Aorto-Coronary Dissection: A Rare Complication With Fatal Prognosis

**DOI:** 10.7759/cureus.60690

**Published:** 2024-05-20

**Authors:** Hema Pamulapati, Siva Sagar Taduru, Pramod Janga, Ajay Kumar Kaja

**Affiliations:** 1 Cardiovascular Disease, University of Kanas Medical Center, Kansas City, USA; 2 Cardiology, Hays Medical Center, Hays, USA; 3 Cardiovascular Medicine, University of Kansas, Kansas City, USA; 4 Internal Medicine, Hays Medical Center, Hays, USA; 5 Cardiology, Piedmont Heart Institute, Fayetteville, USA

**Keywords:** arterial dissection, left heart cardiac catheterization, iatrogenic coronary dissection, iatrogenic aortic dissection, cardiac catheterization complications

## Abstract

Iatrogenic aorto-coronary dissection (IACD) is a rare complication of interventional and surgical cardiac procedures, with a very high mortality burden. Here, we report the case of a 71-year-old female with a past medical history of paroxysmal atrial fibrillation, mild to moderate aortic insufficiency, hypertension, and hyperlipidemia, who presented with classic anginal symptoms and underwent a cardiac catheterization, during which she suffered Iatrogenic right coronary artery (RCA) dissection and ascending aortic dissection resulting in sudden death. IACD is a rare complication, with a fatal prognosis. Coronary angiography and percutaneous coronary intervention (PCI) are considered safe, with a low risk of major complications including coronary perforations, and a very low risk of Iatrogenic aortic dissection (IAD). The coronary injury occurs more commonly during PCI of chronic total occlusion (CTO) or RCA interventions and can extend to the aortic root. IAD is often fatal and has worse outcomes than spontaneous dissection.

## Introduction

The number of coronary angiograms and percutaneous coronary interventions (PCIs) is on the rise, with an estimated 1.02 million cases performed annually in the United States [1]. Alongside the increase in the number of cardiac catheterizations, there has been an increase in case complexity over the years. These procedures are generally considered safe, and the risk of major complications is low, around 0.082% of procedures [1]. Among those, iatrogenic aortic dissection (IAD) can occur in 0.02% to 0.07% of the procedures. The incidence during cardiac surgical procedures is noted at 0.07%, while transcatheter aortic valve replacement (TAVR) procedures report a slightly higher incidence of 0.10% [2]. This is a rare complication and can lead to adverse outcomes. Dissection can be limited to a coronary artery or extend into the aorta. If the dissection is minimal and the blood vessel is patent with good flow, conservative treatment is recommended. If not, stent implantation at this stage would close the flap and restore blood flow satisfactorily [3,4]. Dunning et al. published a case series of nine patients in 2002, with iatrogenic coronary dissection extending into the aorta. They proposed a classification, with three grades of dissection. Type 1 - dissection limited to sinuses of Valsalva, type 2 - dissection of the ascending aorta outside of the sinuses (4 cm), and type 3 - dissection >/= 4 cm. According to these authors, stent placement was adequate for the limited forms, while surgery was necessary for patients with type 3 [3,5]. The reported mortality rate for surgically managed IAD, in conjunction with heart surgery is almost double that of surgically managed spontaneous aortic dissection [6]. Early mortality due to cardiogenic shock was the main reason for mortality in these cases [7,8].

## Case presentation

A 71-year-old female with a past medical history of paroxysmal atrial fibrillation, mild to moderate aortic regurgitation, moderate tricuspid regurgitation, hypertension, hyperlipidemia, and severe obstructive sleep apnea presented to our office for evaluation of chest pain. She complained of substernal chest pressure that had been lasting for about 10 min, occurring with exertion and relieved by rest. She noted that the episodes were occurring more frequently, about once or twice a week. She also complained of mild pedal edema. An electrocardiogram (EKG) in the office showed sinus bradycardia at 51 bpm and a right bundle branch block. Given her symptoms, she was referred for a cardiac catheterization and echocardiogram. Echocardiogram showed normal LV systolic function with ejection fraction in the 60% to 65% range, moderate to severe left atrial dilatation, mildly dilated right atrium, mild mitral regurgitation, mild to moderate aortic regurgitation and moderate tricuspid regurgitation. She subsequently underwent a coronary angiogram, which showed mild plaquing in the left anterior descending (LAD) coronary artery and left circumflex (LCX). There was a small second marginal branch that had a 60%-70% lesion. The right coronary arteriogram revealed a 99% mid-right coronary artery (RCA) lesion. Distal RCA had about 80% lesions. A 6 French, FR4 guide was placed in the RCA and then suddenly dissection in the RCA was noted, which extended into the aortic root and ascending aorta. The procedure was aborted, and cardio-thoracic surgery was called. The plan was for emergent CT angiography and repair of the dissection. However, the patient quickly became unstable with a drop in blood pressure and pulseless electrical activity (PEA). Cardiopulmonary resuscitation (CPR) was started and despite best efforts patient could not be saved. Dissection extended down to the ascending and descending aorta and the patient passed away before being taken for surgical intervention. Limited echocardiogram obtained during resuscitation, did not reveal any significant effusion or tamponade. In these cases, the sudden hemodynamic instability could be due to significant coronary and cerebral mal-perfusion due to extensive dissection, aortic rupture, aortic insufficiency, and sudden heart failure in addition to tamponade.

**Video 1:** Left coronary angiogram

**Video 2:** Right coronary angiogram showing severe stenosis

**Video 3:** Right coronary angiogram showing aorto-coronary dissection

**Video 4:** Aorto-coronary dissection

## Discussion

Iatrogenic aorto-coronary dissection (IACD) following interventional procedures is rare and prognostic information is limited [9]. Type A aortic dissection is associated with significant mortality. Epidemiologic characteristics of IAD differ from spontaneous aortic dissection. Consequently, older patients, those with diabetes mellitus, hypertension, a higher degree of atherosclerosis, and previous aorto-coronary bypass, were more likely to experience iatrogenic dissections. Despite surgical management, iatrogenic forms have been found to have a worse prognosis and greater mortality rate than spontaneous forms (International Registry of Aortic Dissection). In a retrospective study analyzing 74 patients with aortic dissection after interventional procedures, the incidence of IAD was 0.06% [9]. 63.5% of these patients had a dissection while trying to engage the RCA. The incidence of catheter-induced coronary artery dissection (CICAD) is low at <0.1%, although this could have been under-reported [10]. In CICAD, there is disruption of the endothelial layer, with extravasation of blood into sub-endothelial tissue planes [10]. The National Heart, Lung, and Blood Institute (NHLBI) classified CICAD into six types (A to F) based on their angiographic appearance [10,11]. Type A is the mildest form of catheter-induced coronary dissection, limited to the intima. In Type A dissection, there are radiolucent areas during contrast injection with no persistence of contrast. Type B dissections extend beyond the intima and into the media. These dissections show parallel tracts, separated by a radiolucent area during contrast injection, with no significant dye persistence after injection. Type C dissections are more extensive, extending into the adventitia (outer layer) of the coronary artery. These dissections have contrast outside the coronary lumen with the persistence of contrast after the dye has cleared from the coronary lumen. Type D dissections can lead to impaired blood flow, with spiral filling defects and excessive dye staining of the false lumen. Type E dissections may extend into multiple coronary arteries and show persistent filling defects in the coronary lumen. Type F dissections are the most severe form and may lead to complete occlusion of the coronary lumen, impeding antegrade flow. These dissections may propagate retrograde and involve the aorta.

The incidence of CICAD is low, estimated to be less than the 0.1% range, although the true frequency is likely under-reported [12]. In a 10-year retrospective study, with 56,968 patients undergoing coronary angiography, half of the cases of iatrogenic catheter-related dissections occurred more frequently in the RCA, with most of them caused by guiding catheters [13]. Many of these were managed with stenting (82%), some with conservative management (12%), and about 6% requiring surgery [13]. Antegrade dissections are more common than retrograde dissections extending into the aorta.

If the dissection is limited to the coronary artery and the dissection flap is localized with the patent vessel and thrombolysis in myocardial infarction 3 (TIMI 3) flow with complete perfusion, conservative management may be reasonable. If there is a flow-limiting coronary dissection or acute closure of the artery, urgent revascularization is recommended to prevent injury to the myocardial territory. The extent of dissection, flow in the distal bed, hemodynamic stability, and presence of ischemia guide further management in these cases. Retrograde dissection into the aorta can be contained sometimes by stenting and sealing the entry site of the dissection [14]. Emergent ascending aorta replacement is advised when the dissection extends more than 40 mm from the coronary artery ostium, or if the patient presents with severe aortic insufficiency, hemopericardium, or is hemodynamically unstable. In certain scenarios, stenting can serve as a temporary solution to manage the dissection before surgical intervention, helping to stabilize the patient and possibly reduce the progression of the dissection. Extensive IAD often necessitates surgical intervention, primarily involving the replacement of the ascending aorta and possibly the arch, with a significant portion of patients undergoing these extensive procedures. Early mortality remains alarmingly high, underscoring the severity of this complication.

## Conclusions

In summary, published data remain too limited, particularly regarding aortic dissections occurring from interventional procedures. Increased risk of aorto-coronary dissection is linked to factors such as unusual coronary anatomy, the presence of atherosclerosis, stiffer guidewires, catheter curvature, and extensive catheter manipulation. Dissection is seen more often in RCA catheterization than left main coronary artery, due to variations in anatomical and histological features. Overall, acute aorto-coronary dissection is a life-threatening condition that needs prompt diagnosis and management. Depending on the extent of the dissection and hemodynamic stability, management and outcomes vary significantly.
